# Complete Genomes of *Theileria orientalis* Chitose and Buffeli Genotypes Reveal within Species Translocations and Differences in ABC Transporter Content

**DOI:** 10.3390/pathogens11070801

**Published:** 2022-07-15

**Authors:** Jerald Yam, Daniel R. Bogema, Melinda L. Micallef, Steven P. Djordjevic, Cheryl Jenkins

**Affiliations:** 1 NSW Department of Primary Industries, Elizabeth Macarthur Agricultural Institute, Menangle, NSW 2568, Australia; jerald_yam@nea.gov.sg (J.Y.); daniel.bogema@dpi.nsw.gov.au (D.R.B.); melinda.micallef@dpi.nsw.gov.au (M.L.M.); 2 Australian Institute for Microbiology & Infection, University of Technology Sydney, Ultimo, NSW 2007, Australia; steven.djordjevic@uts.edu.au

**Keywords:** *Theileria orientalis*, comparative genomics, gene presence

## Abstract

*Theileria orientalis* causes losses to cattle producers in Eastern Asia, Oceania and, more recently, North America. One pathogenic genotype (Ikeda) has been sequenced to the chromosomal level, while only draft genomes exist for globally distributed Chitose and Buffeli genotypes. To provide an accurate comparative gene-level analysis and help further understand their pathogenicity, we sequenced isolates of the Chitose and Buffeli genotypes of *T. orientalis* using long-read sequencing technology. A combination of several long-read assembly methods and short reads produced chromosomal-level assemblies for both Fish Creek (Chitose) and Goon Nure (Buffeli) isolates, including the first complete and circular apicoplast genomes generated for *T. orientalis*. Comparison with the Shintoku (Ikeda) reference sequence showed both large and small translocations in *T. orientalis* Buffeli, between chromosomes 2 and 3 and chromosomes 1 and 4, respectively. Ortholog clustering showed expansion of ABC transporter genes in Chitose and Buffeli. However, differences in several genes of unknown function, including DUF529/FAINT-domain-containing proteins, were also identified and these genes were more prevalent in Ikeda and Chitose genotypes. Phylogenetics and similarity measures were consistent with previous short-read genomic analysis. The generation of chromosomal sequences for these highly prevalent *T. orientalis* genotypes will also support future studies of population genetics and mixed genotype infections.

## 1. Introduction

*Theileria orientalis* is a haemoprotozoan parasite transmitted by ticks and has a global distribution [1]. Oriental theileriosis caused by the pathogenic genotype Ikeda (Type 2) has been reported in many countries including Australia [2], New Zealand [3], Japan [4] and recently, the USA [5]. Only occasionally has the Chitose genotype been associated with disease [3,6,7], in the presence or absence of the Ikeda genotype, while the Buffeli genotype is almost always benign [7,8,9]. The parasite has often been referred to individually as *T. orientalis*, *T. sergenti*, *T. buffeli* or as a species complex [10,11]. Nomenclature difficulties have been due to various factors including the common occurrence of mixed infections, similar strain morphology and variability of host and parasite interactions [9,12]. More recent investigations have revealed 11 genotypes [1,13,14] and the species is now generally cited as *T. orientalis*. Taxonomic classification of *T. orientalis* has more recently revolved around phylogenetic analysis of the 18S rRNA, p23 and, most notably, the major piroplasm surface protein (MPSP) genes [1,2,15]. The difference in pathogenicity between genotypes and consistent observations of multi-genotype infections mean that correct taxonomic classification is important to differentiate *T. orientalis* genotypes and achieve accurate identification. However, the concurrent infection of several genotypes with similar sequence but varying pathogenicity also creates difficulties for genomic epidemiology and further understanding could be gained through high-throughput sequencing (HTS) technologies.

Several large-scale studies have demonstrated the utility of HTS to improve understanding of phylogenetics, epidemiology and population structure of protozoan parasites [16,17]. The first complete *T. orientalis* genome [18] was achieved with Sanger sequencing and of the Ikeda genotype (strain Shintoku) in 2012. Since then, three additional Australian-sourced isolates were sequenced using the Illumina platform to generate fragmented draft genomes of genotypes Ikeda (Robertson strain), Chitose (benign subtype B;Fish Creek strain) and Buffeli (Goon Nure strain) [19]. However, excluding these, almost no HTS studies of *T. orientalis* have been published in a decade since the first genome sequence was produced. One reason for this may be the difficulty in examining *T. orientalis* genomes due to the presence of mixed infections. To help overcome this, we have generated the first chromosomal assemblies of *T. orientalis* Chitose and Buffeli genotypes. These assemblies were constructed in a hybrid manner, combining Illumina short reads [19] with Oxford Nanopore long reads. Construction of these assemblies has allowed us to further resolve the gene and chromosomal structure of *T. orientalis* Chitose and Buffeli genotypes. The availability of reference assemblies for these genotypes will enable future studies of mixed infections and population genetics.

## 2. Results

### 2.1. Sequencing and Chromosomal Assembly Metrics

Assembly metrics were calculated using *T. orientalis* Shintoku as a reference (Table 1). Approximately 433× and 104× mean coverage depth was achieved for the Fish Creek (Chitose) and Goon Nure (Buffeli) isolates, respectively. The GC content of both isolates was lower than that of Shintoku (see Table S1), which was consistent with the previous short-read study [19]. Raven and Shasta produced assemblies with the lowest number of contigs and highest average N50 values.

Long-read assembly is still a developing technology and different algorithms often perform better at assembling different regions of a chromosome [20]. To address this, we performed draft long-read assembly with five different assembly algorithms and merged draft assemblies with Trycycler. The Trycycler pipeline clearly identified four contig clusters of similar size to the four chromosomes of *T. orientalis* Shintoku. Other contig clusters identified by Trycycler represented fragment contigs of chromosomal and non-nuclear *T. orientalis* DNA. Additional contaminant contigs were identified in these clusters sourced from *Bos taurus* or bacterial sequences and were removed from the final analysis. The four largest clusters were selected for further reconciliation, alignment, partitioning and consensus steps of the Trycycler pipeline and further polished to generate final merged assemblies (Table 2).

### 2.2. Apicoplast Genomes

**Figure 1:**
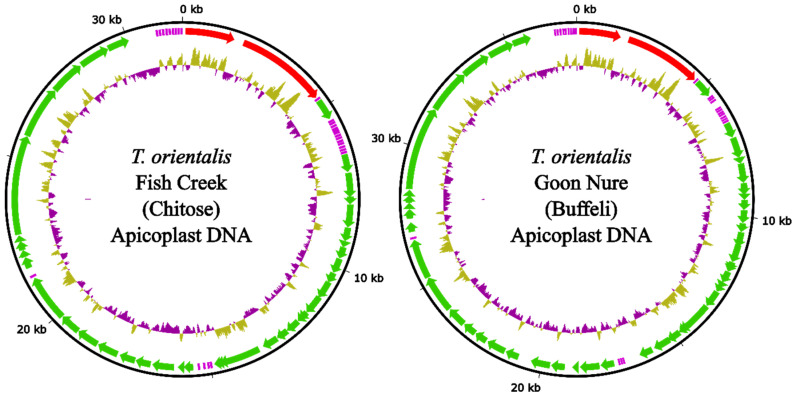
Apicoplast genomes of *T. orientalis* Fish Creek and Goon Nure isolates. Outer ring (black) represents DNA sequence. Middle ring shows annotated genes including ribosomal RNA subunits (red), transfer RNA (purple) and protein coding sequences (green). Inner ring shows %GC difference from average with a 100 bp sliding window.

The apicoplast genome of the Fish Creek isolate is 31.7 kbp in size with a total of 38 protein coding and 26 tRNA genes. The Goon Nure isolate has a slightly larger apicoplast genome at 37.5 kbp with a total of 45 protein coding and 24 tRNA genes (Figure 1). Both genomes contain small- and large-subunit rRNA, and genome synteny is broadly consistent with *T. parva.* Areas of major difference to *T. parva* include the deletion of 10 hypothetical protein genes located between RNA polymerases *rpoC1* and *rpoC2.1* in both Fish Creek and Goon Nure. Fish Creek and Goon Nure isolates also contain a large insertion of hypothetical genes between the *tufA* elongation factor Tu and the *clpC* chaperone genes. The number of protein coding genes in this region increases from three in *T. parva* to eight and fourteen in Fish Creek and Goon Nure, respectively. Comparison with the *T. orientalis* Shintoku apicoplast sequence reveals deletion of the apicoplast ribosomal protein L5 gene. However, the *tufA* → *clpC* genomic region that shows the highest variation between Fish Creek and Goon Nure is missing from the partial Shintoku sequence. Transfer RNA genes are present in three clusters plus two singleton genes and are mostly consistent between *T. orientalis* isolates, with the addition of two tRNA genes in Fish Creek consisting of a second copy of Gln-TTG as well as an intron-containing Lys-TTT gene, also found in *T. parva*.

### 2.3. Synteny between T. orientalis Genotypes

**Figure 2:**
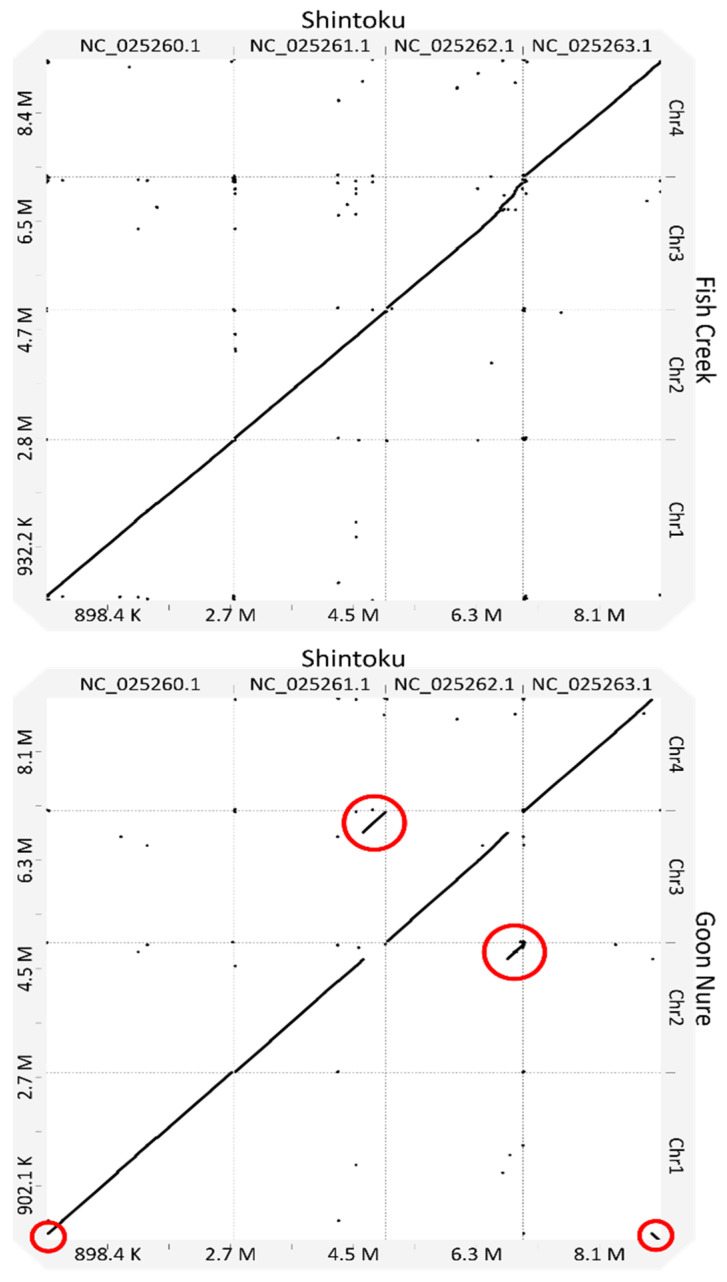

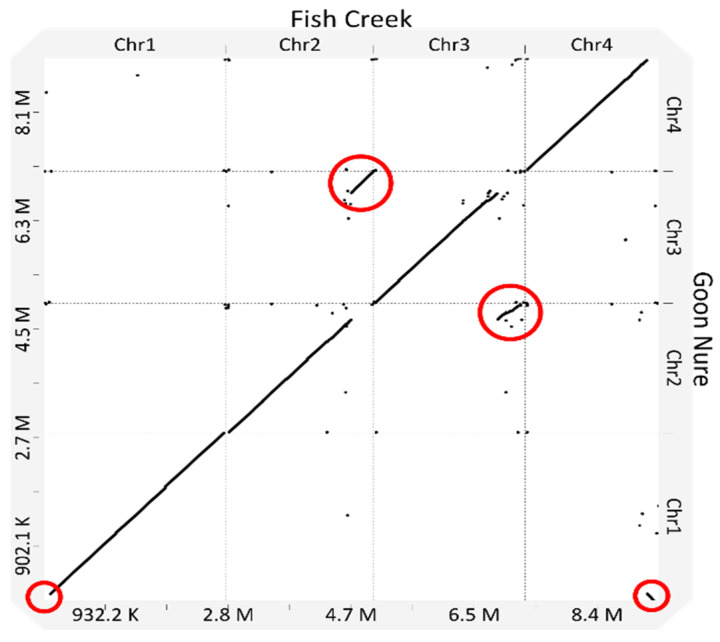
Synteny dot plots of the *T. orientalis* Shintoku (Ikeda) reference and strains Fish Creek (Chitose) and Goon Nure (Buffeli). Red circles indicate rearrangement in strain Goon Nure between chromosomes 2 and 3 and translocation between chromosomes 1 and 4.

When compared to the Shintoku reference, size differences were observed in several chromosomes across both Chitose and Buffeli genotypes (Table 2). The length of chromosome 3 in the Fish Creek isolate is larger than that of both Shintoku and Goon Nure. Chromosome 4 of both Fish Creek and Goon Nure is smaller than the respective chromosome 3, with the opposite observed in Shintoku. Nucmer alignments of Goon Nure chromosomes against their Shintoku equivalents show two chromosomal translocations between chromosomes 2 and 3 (Figure 2) and chromosomes 1 and 4. Additionally, Fish Creek chromosome 3 shows much lower sequence homology and insertion of sequence at the 3′ end of the molecule.

### 2.4. Ortholog Clustering

**Figure 3:**
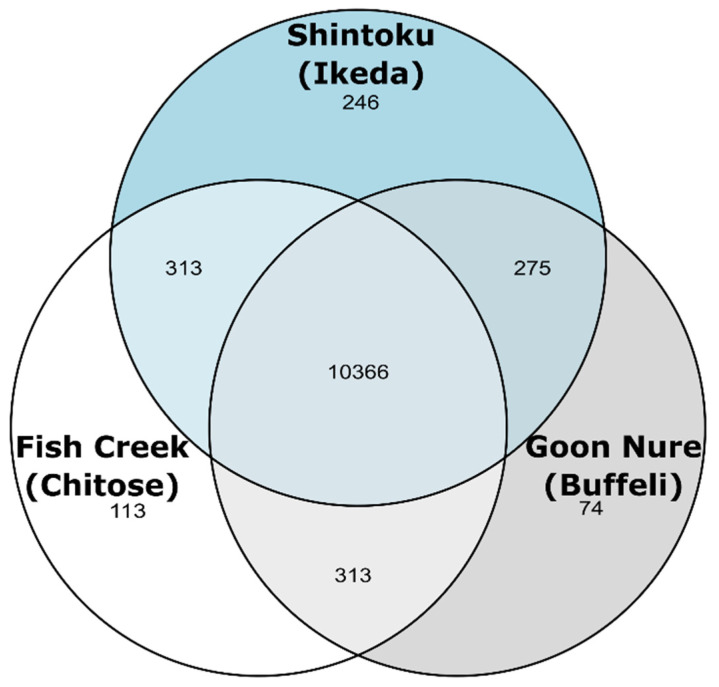
Venn diagram showing number of genes found in each isolate combination.

Evidence-based annotation produced 3980 and 3924 genes in the Fish Creek and Goon Nure strains, respectively, as compared to 4058 genes identified in the Shintoku reference annotation (Table 3). Ortholog clustering of the cumulative 11,757 coding genes showed that 10,366 had orthologs in all three isolates and 901 had orthologs in two (Figure 3). A total of 246, 113, and 74 genes were identified as being unique to the Shintoku, Fish Creek, and Goon Nure isolates, respectively.

**Figure 4:**
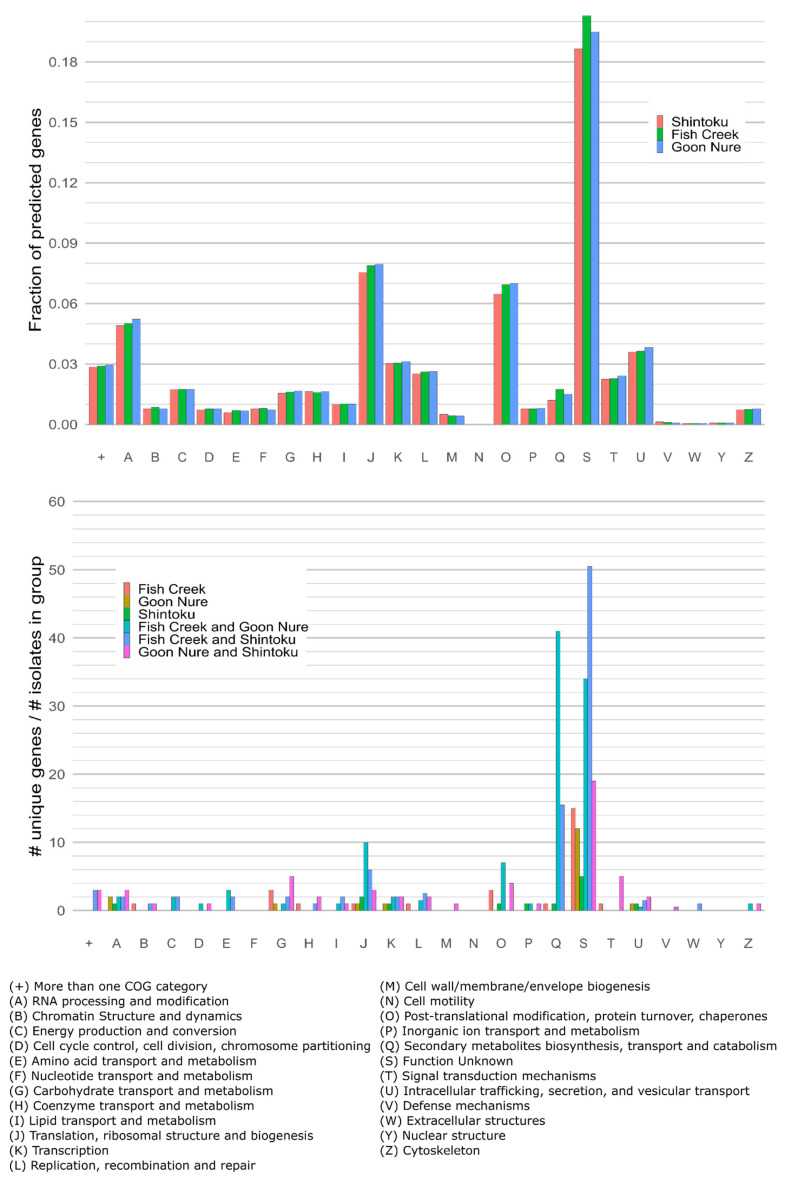
COG analysis of all predicted genes (**top**); genes identified as unique to each isolate combination (**middle**). Genes without COG assignment are not shown but consist of 31–38% of the total gene content of each isolate. COG categories (x-axis) are summarised by their letter categories (**bottom**).

Clusters of Orthologous Groups (COGs) analysis of the entire gene content revealed only minor differences in gene content between *T. orientalis* isolates (Figure 3). The gene content of each isolate comprised approximately 31–38% genes that were not categorised by COG analysis. Comparison of genes only present or absent in one type (i.e., orthologs that clustered to groups containing less than three isolates) shows the most substantive differences in COG categories Q and S (Figure 4). High numbers of category Q (Secondary metabolite biosynthesis, transport and catabolism) genes found in Chitose and Buffeli type isolates were not found in Ikeda, while a smaller number of genes found in Ikeda and Chitose isolates were not found in Buffeli. Few genes of this category were found in a single isolate. Similarly, category S (Function unknown) genes were much more often found to be missing in a single isolate. Observed increases in post-translational modification, protein turnover, and chaperone genes (category O); and translation, ribosomal structure and biogenesis genes (category J) were found in Chitose and Buffeli isolates. However, these categories were artificially inflated by the presence of full-length apicoplast sequences in these isolate assemblies, as the apicoplast is rich in ribosomal proteins (J) and others such as ATP-dependent Clp protease (O).

Further examination of unique gene categories reveals that almost all category Q genes unique to both the Fish Creek and Goon Nure isolates cluster into two orthogroups (N8.HOG0000000 and N8.HOG0000001) containing a substantial number of genes with ATP-binding cassette (ABC) transporter domains (Table S2). BLASTP analysis of these genes against the nr database limited to *Plasmodium* hits showed that most of these predicted proteins have moderate similarity to multidrug resistance proteins MDR1 and MDR2. Examination of sequence annotations identified a total of 38 genes in Ikeda isolate Shintoku containing the ABC transporter Pfam identifiers PF00005 and PF00664, whereas isolates Fish Creek (Chitose subtype B) and Goon Nure (Buffeli) contained a total 55 and 43 ABC transporter genes, respectively, with 43 and 36 of these unique to benign isolates.

Analysis of unique category S genes and those not categorised in COG analysis of unique genes present in a single isolate revealed several differences. In Shintoku, most unique genes (n = 195) did not cluster into orthogroups. These genes were almost exclusively encoded hypothetical proteins, with only 2/195 proteins matching Pfam domains, and were generally smaller in size when compared with genes that clustered with other Shintoku proteins (median protein length = 57 vs. 270). Unique predicted Shintoku proteins that formed orthogroups with other Shintoku proteins showed a greater number of hits in annotation databases. The largest orthogroup (N8.HOG0000481) consisted of 13 hypothetical proteins predicted to have a single C-terminal transmembrane domain and matched EggNog ID ENOG502RZ20. A similarly annotated group of proteins were detected as unique in Fish Creek (N8.HOG0000334) and to a lesser extent in Goon Nure (N8.HOG0003233). The similarity of these annotations (and gene locations) is interesting considering they cluster well within genotypes but do not cluster between genotypes. Unique predicted proteins of category S in Fish Creek and Goon Nure primarily contained the DUF529 or FAINT protein domain previously identified in this genus [18]. This domain was found in 12 proteins unique to Fish Creek and 6 unique to Goon Nure, compared with only 1 FAINT protein unique to Shintoku.

One notable observation from ortholog clustering results is that the benign isolates share several orthogroups with a high number of genes (Table S2). The Fish Creek/Goon Nure isolates share 11 orthogroups of greater than three genes, whereas only 5 are found in the Fish Creek/Shintoku pair and none for Goon Nure and Shintoku. These larger ortholog clusters generally fell into several categories including ABC Transporter genes, DUF529/FAINT-domain-containing proteins, *Theileria*-associated proteins of unknown function (EggNog OG—ENOG503KDY7) and hypothetical proteins containing predicted transmembrane domains. Shintoku and Fish Creek share the highest number of unique category S (Function unknown) genes; however, the majority of these (88/101) are in orthogroups of three genes or less. FAINT-domain proteins are highly represented in *Theileria* spp. and while Fish Creek contains similar numbers of proteins containing the FAINT-domain (145) as the Shintoku sequence (142), Goon Nure contains a lower number (117). Additionally, a previously identified member of the TashAT cluster of transforming *Theileria* spp. in *T. orientalis* also appears to be single copy within Fish Creek and Goon Nure.

### 2.5. Phylogeny and Average Nucleotide Identity

**Figure 5:**
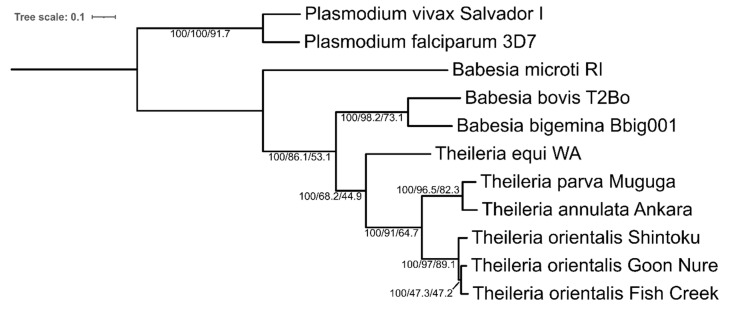
Maximum likelihood tree of Piroplasmida whole-genome protein sequences inferred with concordance factors with IQ-TREE 2 using 1417 concatenated protein sequences from single-copy genes. *P. vivax* str. Salvador I and *P. falciparum* str. 3D7 were used as outgroups. Each branch label on the tree shows the bootstrap, gene concordance factor (gCF) and site concordance factor (sCF), respectively (bootstrap/gCF/sCF).

To confirm species relationships observed previously [19], a phylogenetic tree was inferred by maximum likelihood using an alignment of 848 concatenated protein sequences from single-copy genes (Figure 4). Bootstrap support values were 100% across all branches. Gene concordance (gCF) and site concordance factors (sCF) showed lower values in some branches. The branch containing Chitose (Fish Creek) and Buffeli (Goon Nure) isolates shows low concordance, with more than half of gene trees showing alternate tree orientations, which is either Fish Creek or Goon Nure sharing a branch with Shintoku (Figure 5). Examination of gene trees discordant with the species tree at this branch showed 24.1% and 27.0% of gene trees with Ikeda/Chitose and Ikeda/Buffeli pairings, respectively. A chi-squared test shows these numbers are not significantly different (P = 0.126), indicating this may be due to incomplete lineage sorting [21,22]. Average nucleotide identity (ANI) of the *Theileria* genus was also explored to compare genetic relatedness using whole-genome sequences (Figure S1). Results from this analysis are similar to the previous study [19], with pairwise identities between Ikeda and Chitose/Buffeli isolates ranging from 81.7 to 82.0% and Chitose and Buffeli isolates showing an average identity of 85.4%. Separate species *T. annulata* and *T. parva* (79.5%) show a difference similar to the Ikeda vs. Chitose/Buffeli comparison.

## 3. Discussion

In this study, we explored a multi-assembler approach to complete chromosome-level assemblies missing for two of the major *T. orientalis* genotypes found in Australian cattle. To combine multiple assemblies, we used Trycycler, a pipeline designed for bacterial sequences that compares and merges sequences from multiple long-read assembly methods to generate a more contiguous consensus [20], which can then be corrected further by polishing with short- and long-read data. The availability of the *T. orientalis* Shintoku sequence allowed us to compare each method for accuracy and contiguousness. Some assembly algorithms were able to assemble genome regions that others could not, allowing for assemblies to be merged to produce chromosome-level assemblies. Each chromosome was constructed from, at minimum, two independent assembly methods.

In our previous study of *Theileria orientalis* Chitose (Fish Creek) and Buffeli (Goon Nure) genomes using short-read sequencing, we identified low pairwise ANI values when compared with the Shintoku reference and an Australian-sourced Ikeda genome (Robertson) [19]. When updated with full genome sequences generated in this study, comparisons of Fish Creek and Goon Nure with the Shintoku reference sequence show slightly lower ANI values than previously observed (81.7% vs. 82.5%) [19]. As previously indicated, this number is comparable with the ANI observed between *T. parva* and *T. annulata* (79.6%), which was consistent in both studies [19]. Genome-wide phylogenetic relationships with high bootstrap support were also consistent between this study and the previous, with Chitose/Buffeli sharing a recent common ancestor and Ikeda separating from these strains prior [19]. While bootstrap values were high for this topology, gene and site concordance were lower than other tree branches potentially due to incomplete lineage sorting.

In the Goon Nure isolate, we observed a chromosomal translocation between chromosomes 2 (260 to 280 kbp) and 3 (1.825 to 1.845 mb). This translocation was consistent across all assembly methods. To further examine this region, long reads were mapped back to the final hybrid assembly and manually inspected. There were several long reads spanning breakpoints and no evidence indicating misassemblies of any of the four assembly methods. The translocation of a section of chromosome 4 to chromosome 1 was also supported with high read coverage. Genome synteny is highly conserved within *Babesia* and *Theileria* genera [23]. Additionally, in the genus *Plasmodium*, conservation of synteny follows phylogenetic patterns with similar species sharing genome arrangement [23]. Comparison of *T. parva* and *T. annulata* genomes shows one major and two minor intrachromosomal rearrangements on chromosome 3 and a very small (5 gene) interchromosomal translocation between chromosomes 1 and 4. The presence of multiple interchromosomal translocation events in the Buffeli genotype is interesting and further sequencing of this genotype in overseas isolates would identify if this is a common occurrence or restricted to Australian populations.

Here, we have sequenced the first complete and circular apicoplast genomes of *T. orientalis*. The *T. orientalis* apicoplast genome structure is largely consistent with previously sequenced *T. parva* with a highly similar genetic organisation identified [24]. Comparison with the published Shintoku sequence revealed small differences, but conclusions were limited by the lack of a complete apicoplast genome for *T. orientalis* Ikeda. Apicoplast genes are often considered high-value targets for therapeutics due to genetic and functional differences caused by their bacterial ancestry [25]. Further work to complete an Ikeda-sourced apicoplast genome could reveal further therapeutic targets.

COG analysis showed few functional genetic differences between these isolates, which is likely linked to their close phylogenetic relationship. The clearest difference between the gene content of these isolates was in the number of Q and S category genes found in orthogroups unique to a combination of one or two isolates (Figure 4). The benign Chitose subtype B and Buffeli isolates contained a higher number of these genes when compared with the pathogenic Ikeda isolates. Further analysis of the Q category showed larger differences in the number of ABC transporter genes. ABC transporters are very large superfamily of proteins present in all known living organisms and provide membrane translocation for a diverse range of substrates. These genes have been implicated in multidrug resistance in several organisms but have significant additional functions. The number of these genes could be associated with a difference in virulence as gene knockouts of one ABC transporter in *P. falciparum* have been clearly associated with increased gametocyte formation [26]. Conversely, removal of *mdr1* and *mdr2* genes appear to have little effect on the sexual and asexual stages of *P. falciparum* but are critical for development within hepatocytes [27]. Removal or reduction in the number of these genes may have different effects in *T. orientalis*, where the life cycle does not include infection of hepatocytes [28]. In other *Theileria* spp., ABC transporter gene number can vary substantially; *T. equi* also contains a large repertoire of ABC transporter genes (45 in total), while highly pathogenic *T. parva* contains only 17 [29].

## 4. Materials and Methods

### 4.1. Sample Collection and Processing

*T. orientalis* Fish Creek and Goon Nure strains were confirmed as single-genotype infections, sourced, sampled, propagated in splenectomised cattle and purified from cattle blood as previously described [19].

### 4.2. Illumina Sequencing

Genomic DNA was previously extracted from purified piroplasms using the DNeasy blood and tissue kit (QIAGEN) [19]. Illumina sequences of the *T. orientalis* genotypes Chitose (subtype B) [30] and Buffeli were generated previously using MiSeq V2 chemistry to produce 250 bp paired-end reads and the HiSeq2500 system to produce 150 bp paired-end reads [19].

### 4.3. Genomic DNA Extraction

Technical replicates of purified piroplasms sequenced by the Illumina method above were stored at −80 °C at the Elizabeth Macarthur Agricultural Institute (EMAI) prior to extraction. These isolates were confirmed to be of a single genotype by two different genotyping PCRs [4,19,31]. Genomic DNA was extracted from purified piroplasms using a modification of the Qiagen Genomic-tip DNA kit (Qiagen) procedure, as follows. A 7 µL aliquot of 100 mg/mL RNase A solution was added to 3.5 mL of buffer B1. The purified piroplasm pellet was resuspended in the RNase A–buffer B1 solution by vortexing and then 1 mL of 20 mg/mL of proteinase K was added. The samples were incubated at 37 °C for 30 min and shaken at 150 rpm. Buffer B2 (1.2 mL) was added to the samples which were mixed by 10 inversions and transferred into a hybridisation oven for incubation at 56 °C overnight with rotation. The following day, samples were centrifuged at 5000× *g* at 4 °C for 10 min and the supernatant was retained. Buffer QBT (4 mL) was added to a Genomic-tip for equilibration and allowed to drain completely by gravity flow. Samples were vortexed for 10 s at maximum speed and transferred to the equilibrated Genomic-tip column. The column was then washed twice with 7.5 mL of buffer QC. The genomic DNA sample was eluted with 5 mL of pre-warmed (50 °C) buffer QF. To precipitate the DNA, 3.5 mL of isopropanol was added to the eluted genomic DNA and incubated overnight at room temperature. The following day, the genomic DNA samples were mixed and centrifuged at 10,000× *g* for 15 min at 4 °C in 2 mL aliquots in 2 mL centrifuge tubes (two tubes each for each isolate). The supernatant was removed, and each pellet was washed with 2 mL of cold 70% ethanol. Samples were briefly vortexed and centrifuged at 10,000× *g* for 10 min at 4 °C. The supernatant was carefully removed and allowed to air dry for 10 min. The pellets were each resuspended in 50 µL of Tris-HCl (pH 8) and pooled and allowed to redissolve overnight at room temperature. The concentration of the genomic DNA samples was measured using the Qubit 3.0 fluorometer (Life Technologies, Carlsbad, CA, USA) and the quality of the DNA was measured with the NanoVue spectrophotometer (GE Life Sciences).

### 4.4. Pulsed-Field Gel Electrophoresis (PFGE)

PFGE analysis was performed to determine fragmentation of the genomic DNA samples. As smaller fragments are preferentially sequenced by Oxford Nanopore devices, the identification and removal of small fragments (<2 kb) are critical for efficient sequencing of long reads needed for genome scaffolding. DNA samples were added to a 1% SeaKem Gold agarose (Lonza, Basel, Switzerland) gel and transferred into the electrophoresis tank. PFGE was performed under these conditions: 6 V/cm voltage, 5 to 15 s switch time, 4 h run time at 14 °C. PFGE gel was stained with GelRed (Biotium, Fremont, CA, USA) for 10 min on a low-speed shaker and de-stained in distilled water for 10 min. The gel was analysed on the Gel Doc XR+ (Bio-Rad, Hercules, CA, USA).

### 4.5. Nanopore Library Preparation and Sequencing

Post PFGE analysis, libraries were prepared from genomic DNA samples using the 1D Genomic DNA ligation protocol (SQK–LSK108, Oxford Nanopore Technologies (ONT), Oxford, UK) according to the manufacturer’s instructions. Genomic DNA samples were sequenced using two R9.4.1 MinION flow cells (ONT) with the MinKNOW software v1.14.1 or v1.15.1 (ONT). The MinION run duration for *T. orientalis* Chitose (Fish Creek) and *T. orientalis* Buffeli (Goon Nure) was 13 h and 26 h, respectively.

### 4.6. Sequence Quality Assessment

Unless otherwise indicated, software for bioinformatic analysis was installed using Bioconda [32]. Following ONT MinION sequencing, all reads were basecalled with guppy_basecaller v6.0.1+652ffd179 (ONT) and adapters were removed using Porechop v0.2.3. Nanopore reads were assessed with PycoQC v2.5.0.3 [33] and FastQC v0.11.7.

### 4.7. Genome Assembly

Long-read assembly was achieved using a combination of five assemblers, which utilise different assembly methodologies, followed by merging with Trycycler [20]. Nanopore reads were first filtered for length and quality using Filtlong. Initial assemblies of chromosomal DNA were built with up to six replicate assemblies for each of the following assemblers: Flye v2.9 [34], Miniasm v0.3, Raven v1.7.0 [35], Necat v0.0.1_update20200803 [36] and Shasta v0.8.0 [37]. Assembly contigs were merged with Trycycler. All relevant Trycycler steps were performed with the -linear option to prevent circularisation of contigs. Consensus contigs were further polished with long reads using Medaka v1.5.0. Further correction of assemblies with previously generated short reads was performed using first Polypolish [38] and then Polca from the MaSuRCA assembler package [39], and repeated until consensus. Completeness of chromosomal assemblies was assessed by aligning to the *T. orientalis* Shintoku genome (NW_009646193.1) with Mauve [40]. To assemble Apicoplast DNA, Nanopore reads were aligned using Minimap2 [41] to a partial Apicoplast reference obtained from the *T. orientalis* Shintoku genome (NW_009646193.1). Mapped reads were extracted using Samtools [42], filtered, assembled, merged, and polished with Trycycler as described above. Full-length mitochondrial sequences were obtained from previous Illumina assemblies [19].

### 4.8. Genome Annotation

Gene prediction and functional annotation were performed with the Funannotate v1.8.9 pipeline. Ab initio gene predictors Augustus v3.3.3 [43], SNAP v2013–02–16 [44], GlimmerHMM v3.0.4 [45] and Genemark-ES v4.68 [46] were trained with “funannotate train” using the Shintoku genome sequence and *T. orientalis* RNA-seq data from the Sequence Read Archive (DRR118936–9). Prior to training, RNA-seq data were quality-controlled and trimmed using fastp [47]. Gene prediction was performed with “funannotate predict” using protein alignment, assembled transcript alignment, and trained ab initio predictors. Protein alignments were performed with Exonerate protein2genome v2.4.0 using a minimum identity of 40% [48]. For transcript alignments, Trinity v2.8.5 [49] assemblies from *T. orientalis* RNA-seq data (generated during training step) were combined with *T. orientalis*-expressed sequence tag sequences (FS565182–6375). The above gene predictors, protein and transcript alignments were assessed with EvidenceModeler v1.1.1 [50] and consensus gene models from all data used to predict genes.

Additionally, predicted genes were added and modified through comparison with the *T. orientalis* Shintoku genome. Genome annotations were transferred to Fish Creek and Goon Nure sequences using RATT [51] and manually assessed against automated annotations using Geneious v2022.1.1. Automated annotations were modified if they differed significantly from transferred annotations and evidence suggested that a transferred annotation was more correct. This evidence included transcript alignments, presence or absence of open reading frames or presence or absence canonical splice sites. Genes identified by RATT but not predicted by the automated annotation pipeline were manually added if evidence for inclusion (as above) existed.

To achieve functional annotation, “funannotate annotate” was used with InterProScan5 [52], called using funannotate iprscan, and eggNOG mapper [53,54]. Ribosomal RNA subunits were predicted with Barrnap v0.9. Annotation metrics were compiled using inhouse python scripts which have been deposited online at https://github.com/bogemad/gas. Annotation of apicoplast and mitochondrial genome sequences were performed manually with Geneious v2020.1.1. Open reading frames were identified and BLASTP was used to functionally annotate using a similarity cutoff of e = 1e–05 and the *T. parva* (NC_007758) apicoplast genome.

### 4.9. Ortholog Clustering, Phylogeny, Gene Presence/Absence and Average Nucleotide Identity

Ortholog clustering was achieved with Orthofinder v2.5.4 [55] using previously generated sequences from *Babesia bigemina*, *Babesia bovis*, *Babesia microti*, *Theileria annulata*, *Theileria equi, Theileria orientalis* and *Theileria parva* [18,29,56,57,58,59,60]. Predicted proteins from *Plasmodium falciparum* 3D7 and *Plasmodium vivax* Salvador I were used as an outgroup [61]. Gene presence/absence comparisons within *T. orientalis* were generated from phylogenetic hierarchical orthogroups and unassigned genes using custom python scripts, which have been deposited online at https://github.com/bogemad/COG_gene_analysis. Single-copy orthologs identified by ortholog clustering were used to generate a maximum likelihood tree with IQ-TREE 2 [21]. This reference tree was inferred with concatenated single-copy genes with 1000 ultrafast bootstraps [62,63]. Model selection with ModelFinder [64] and calculation of concordance factors [21] were also performed using IQ-TREE 2. Additional scripts for curation of *T. orientalis* genomes can be found online at https://github.com/bogemad/theileria_orientalis_complete_genome_scripts.

Finally, to investigate genome relatedness, pyANI v0.2.9 (-m ANIb) [65] was used to calculate average nucleotide identity using six strains from the *Theileria* genus (including genomes in this study).

## 5. Conclusions

One of the motivations of this study was to generate reference whole genome sequences of *T. orientalis* Chitose and *T. orientalis* Buffeli. Gene differences identified here can potentially be a lead for future studies to experimentally examine functional differences. Further, the generation of these reference sequences provides a resource to examine the population genetics of *T. orientalis* more effectively within and between Ikeda, Chitose and Buffeli genotypes. *T. orientalis* infections very frequently present as a mixture of genotypes in a single host animal [30,66,67], and differences in pathogenicity of these types make population genetic analysis difficult. The sequences generated in this study will benefit future studies of mixed infections by improving in silico separation of genotype sequences, enabling better understanding of the complexity of the disease. The availability of chromosomal sequences may also enable future research aimed at elucidating epidemiology of this parasite through population genomic studies, similar to those undertaken on malaria [16].

## Figures and Tables

**Table 1:** Draft assembly results of the five different assemblers trailed.

Genotype	Assembler	Total Contigs	Contigs (≥50 kb)	Total Length	N50	Largest Contig (bp)
Chitose (Fish Creek)	Flye	14–16	7–8	9,344,963	2,171,492	2,745,486
	Miniasm	7–10	5–6	9,559,641	2,254,955	2,765,560
	Necat	4–6	4–6	9,416,796	2,296,410	2,765,760
	Raven	5–6	4	9,365,432	2,296,609	2,770,085
	Shasta	9–12	4–5	9,427,530	2,242,537	2,765,028
Buffeli (Goon Nure)	Flye	20	7–12	9,316,485	1,958,568	2,504,925
	Miniasm	9–14	6–12	9,531,547	1,733,126	2,109,761
	Necat	10–19	10–19	10,547,787	1,967,178	2,912,118
	Raven	9–12	4–6	9,269,508	2,079,080	2,896,450
	Shasta	17	5–7	9,313,556	2,177,669	2,788,592

**Table 2:** Final chromosome lengths (bp) for sequenced *T. orientalis* isolates.

Isolate	Chr 1	Chr 2	Chr 3	Chr 4	Apicoplast	Mitochondria
Shintoku	2,746,313	2,216,979	2,000,793	2,019,511	24,173 *	2595 *
Fish Creek	2,765,963	2,233,854	2,297,733	2,024,851	31,688	6231
Goon Nure	2,785,604	2,153,779	2,196,581	1,884,878	37,498	5965

* incomplete sequence.

**Table 3:** Genome annotation statistics of the *T. orientalis* isolates sequenced in this study and the *T. orientalis* Shintoku reference sequence.

	Shintoku (Ikeda)	Fish Creek (Chitose)	Goon Nure (Buffeli)
Genome			
Total predicted genes	4058	3980	3924
Total predicted mRNA	4002	3907	3848
Total predicted tRNA	47	66	69
Total predicted rRNA	9	7	7
Total predicted CDS	4002	3907	3848
Percentage coding sequence	68.43	68.46	68.73
Total annotated sequence length	9,010,364	9,360,320	9,064,305
Percentage GC	41.53	38.84	37.46
Genes (+tRNA and rRNA)			
Longest gene	26,436	23,559	25,877
Shortest gene	39	24	33
Total gene length	7,386,640	7,385,994	7,214,907
Average gene length	1820	1856	1839
Average gene coding sequence	1541	1640	1619
Gene density (per 10,000 bp)	450.37	425.2	432.91
Percentage coding genes with introns	78.3	76	76.1
Exons			
Total exon length	6,180,198	6,424,419	6,246,171
Total number of exons	16,558	15,809	15,837
Longest exon	11,241	16,092	25,364
Shortest exon	2	3	3
Average exon length	373.2	406.4	394.4
Percentage GC	46.06	43.21	41.89
Introns			
Total intron length	1,206,442	961,575	968,736
Total number of introns	12,500	11,829	11,913
Longest intron	5418	6291	4043
Shortest intron	4	11	11
Average intron length	96.5	81.3	81.3
Average introns per gene	3.1	3	3
Percentage GC	34.12	29.54	27.26
Intergenic regions			
Total intergenic length	1,636,669	1,974,520	1,849,793
Total intergenic regions	4020	3962	3901
Longest intergenic region	9728	8374	18,440
Shortest intergenic region	1	1	1
Average intergenic length	407.1	498.4	474.2
Percentage GC	29.9	29.12	27.86

## Data Availability

Publicly available datasets were analysed in this study. These data can be found at https://www.ncbi.nlm.nih.gov/assembly; with accession numbers: GCF_000740895.1, GCF_000165365.1, GCF_000003225.4, GCF_000342415.1, GCF_000691945.2, GCF_000981445.1, GCA_000165395.2, GCF_000002765.4, GCF_000002415.2. Data generated in this study have been deposited at NCBI under Bioprojects PRJNA325070 (Fish Creek) and PRJNA325071 (Goon Nure). Raw sequence reads generated in this study have been deposited in SRA under accessions SRR20016367 (Fish Creek) and SRR20015857 (Goon Nure). Annotated assemblies have been deposited under GenBank accessions CP056065, CP056066, CP056067, CP056068, CP100322, and CP100323 for *T. orientalis* Fish Creek and CP056069, CP056070, CP056071, CP056072, CP100324, and CP100325 for *T. orientalis* Goon Nure.

## Supplementary Materials

The following supporting information can be downloaded at: https://www.mdpi.com/article/10.3390/pathogens11070801/s1, Figure S1: Pairwise Average Nucleotide Identity (ANI) comparisons of Piroplasmida species.; Table S1: Output and quality metrics for Illumina and Nanopore reads used in this study. Table S2: Ortholog clusters (orthogroups) identified as unique to an isolate combination consisting of four or more genes.
