# Supplementary material for: Complete Genomes of Theileria orientalis Chitose and Buffeli Genotypes Reveal within Species Translocations and Differences in ABC Transporter Content
Source: Pathogens. 2022 Jul 15;11(7):801. doi: 10.3390/pathogens11070801 (PMC9323827; doi:10.3390/pathogens11070801)
Supplement: Supplementary file 1 [file pathogens-11-00801-s001.zip › Figure S1.pdf]

Theileria annulata str. Ankara	*Theileria equi* str. WA	*Theileria orientalis* str. Shintoku	*Theileria orientalis* str. Robertson (Ikeda)	*Theileria orientalis* str. Goon nure (buffeli)	*Theileria orientalis* str. Fish creek (chitose)	
79.56%	70.44%	69.93%	70.02%	69.88%	69.82%	*Theileria parva* str. Muguga
	70.42%	69.97%	70.16%	70.00%	69.95%	*Theileria annulata* str. Ankara
		70.27%	70.06%	70.70%	70.58%	*Theileria equi* str. WA
			99.13%	81.69%	81.94%	*Theileria orientalis* str. Shintoku
				81.67%	81.96%	*Theileria orientalis* str. Robertson (Ikeda)
					85.35%	*Theileria orientalis* str. Goon nure (buffeli)

Figure S1: Pairwise average nucleotide identities of Theileria genomes represented as percentages inferred from pyANI.
